# Glucagon-like Peptide-1 Receptor Agonists: Exciting Avenues Beyond Weight Loss

**DOI:** 10.3390/jcm14061978

**Published:** 2025-03-14

**Authors:** Lalitha Sundararaman, Divakara Gouda, Anil Kumar, Sumithra Sundararaman, Basavana Goudra

**Affiliations:** 1 Department of Anesthesiology, Brigham and Women’s Hospital, 75 Francis St., Boston, MA 02115, USA; lsundararaman@bwh.harvard.edu; 2 Inspira Health Network, 155 Bridgeton Pike ste c, Mullica Hill, NJ 08062, USA; goudad1@ihn.org; 3 Department of Diabetes and Endocrinology, Karnataka Institute of Endocrinology and Research Bangalore, Binnamangala, Stage 1, Indiranagar, Bengaluru 560038, Karnataka, India; r.anil_kumar@yahoo.co.in; 4 Prana Holistic Center for Fertility and Integrated Medicine, 74/198, St’Mary’s Road, Opp. St’ Mary’s Church, Trustpakkam, Abiramapuram, Chennai 600018, Tamil Nadu, India; sumisundar@yahoo.com; 5 Department of Anesthesiology, Sidney Kimmel Medical College, Jefferson Health, 111 S 11th Street, #8280, Philadelphia, PA 19107, USA

**Keywords:** GLPI-A, obesity, type 2 DM, hypertension, Parkinson, renal failure

## Abstract

The last two decades have proffered many remarkable choices in managing type 1 and type 2 diabetes mellitus. Leading the list are glucagon-like peptide-1 receptor agonists (GLP1RAs), the first of which, exenatide, was approved by the FDA in 2005. Two other major classes of drugs have also entered the market: dipeptidyl peptidase-4 (DPP-4) inhibitors, commonly known as gliptins and approved in 2006, and sodium–glucose cotransporter-2 (SGLT-2) inhibitors, with the first approval occurring in 2013. These drugs have revolutionized the treatment of diabetes. Additionally, on the horizon, the once-weekly basal insulin analog insulin icodec and the once-weekly combination of insulin icodec and semaglutide are expected to be available in the future. Beyond glycemic control, GLP1RAs have exhibited benefits in conditions associated with diabetes, including hypertension, dyslipidemia, non-alcoholic steatohepatitis, as well as in neurodegenerative diseases such as Alzheimer’s disease. Additionally, emerging research suggests potential roles in certain types of cancer, infertility, and associative learning. Major cardiovascular events seem to be lower in patients on GLP1RAs. While some evidence is robust, other findings remain tenuous. It is important that clinicians are familiar with current research in order to provide optimal evidence-based care to patients. In the not-too-distant future, there may be a case to prescribe these drugs for benefits outside diabetes.

## 1. Introduction

Glucagon-like peptide-1 receptor agonists (also known as GLP-1 receptor agonists, GLP-1 analogs, GLP1As, and incretin mimetics) are a popular group of medications used in the treatment of type 2 diabetes (T2DM) and obesity. Examples of drugs in this class include tirzepatide, semaglutide, liraglutide, and dulaglutide, with common brand names such as Mounjaro, Wegovy, Zepbound, Saxenda, and Victoza. Notably, tirzepatide differs from other GLP-1 receptor agonists by acting as a glucose-dependent insulinotropic polypeptide (GIP) receptor agonist, making it the first dual-incretin therapy approved for diabetes. The popularity of GLP1As has soared in recent years, and they are increasingly being studied for potential benefits in conditions frequently associated with diabetes such as hypertension, dyslipidemia, and dementia, as well as in other conditions such as polycystic ovary disease and certain types of cancer. This review discusses the current state of research, aiming to inform readers of the evidence should they consider prescribing GLP1As for diabetes with co-existing conditions, emphasizing their mechanistic roles and clinical implications for both metabolic and non-metabolic disorders.

Table 1 and Table 2 summarize major trials evaluating GLP1As, highlighting their effects on both conditions commonly linked to diabetes and obesity, as well as those less commonly linked.

## 2. GLP1As and Hypertension

Diabetics have an increased risk of hypertension in the setting of chronic kidney disease. Hyperglycemia and the resultant glycosuria increase the activity of sodium–glucose cotransport-2 (SGLT-2), causing increased sodium reabsorption in the proximal tubule along with fluid retention [12]. Insulin resistance and hyperinsulinemia cause increased sodium reabsorption. Hyperglycemia directly stimulates the local production of angiotensin II and enhances tissue response to intrarenal renin–angiotensin–aldosterone system (RAAS) activation [13].

In a study recently published in *Nature*, authors Wadjlich and Nowicki state that GLP1As, especially liraglutide, may moderately lower blood pressure (BP) due to increased natriuresis and the inhibition of the renin–angiotensin–aldosterone system in patients with diabetic kidney disease. A paradoxical increase in BP has been observed as a short-term effect of the drug, possibly due to sympathetic stimulation and increased cardiac output [14]. Goud et al. in their review of evidence have corroborated that short-term infusions of GLP1A over a few hours can increase BP due to an increase in heart rate and cardiac output [15].

A 2024 study published in *Hypertension* reported a reduction in BP in obese patients treated with tirzepatide compared to a placebo. This corresponds with results from the earlier SURMOUNT-1 trial, which demonstrated a significant decrease in average 24 h systolic BP, ranging from 7.4 to 10.6 mmHg, with tirzepatide compared to a placebo, as well as a reduction in 24 h diastolic BP. Additionally, heart rates increased by 2.1 to 5.4 beats per minute across the different doses [1]. Overall, most studies have shown that GLP1As primarily lower systolic BP, with a smaller effect on diastolic BP [16]. In a separate analysis of six liraglutide trials, systolic BP was found to decrease by an average of 2.5 mmHg after two weeks of treatment [17].

The decrease in BP observed with liraglutide occurred prior to any significant weight loss, suggesting a potential direct effect of GLP1As. It has been proposed that this BP reduction may be attributed to a vasodilatory effect, increased natriuresis, and/or the inhibition of the renin–angiotensin–aldosterone system. However, the effects of GLP1As on blood pressure can differ in the short term versus the long term. Several studies involving short-term infusion versus administration lasting hours, primarily in healthy adults, have reported either no change or an increase in blood pressure, along with a rise in heart rate and cardiac output.

GLP1As can increase urine and sodium excretion by partially blocking Na+/H+ exchanger 3 (NHE3) in the renal proximal tubule [18]. The activation of cAMP by GLP1As provides benefits to the kidney, such as reducing urine albumin levels, combating oxidative stress, and reducing inflammation in the diseased kidney [19].

## 3. GLP1As and Dyslipidemia

Type 2 diabetes is an insulin-resistant state associated with an increase in free fatty acids, triglycerides, and LDL cholesterol, as well as a decrease in HDL cholesterol. The regulation of cholesterol plays a crucial role in systemic metabolic disorders such diabetes, as well as in central nervous system conditions such as dementia. GLP1A therapy is shown to decrease the hepatic VLDL-TG synthesis rate and reduce hepatic triglyceride content by modulating key enzymes of lipid metabolism and decreasing hepatocyte de novo lipogenesis and β-oxidation. GLP1As can also modulate reverse cholesterol transport, thereby lowering LDL and raising HDL cholesterol [20]. The secondary benefit of lipid metabolism modulation by GLP1As contributes to their cardiovascular protective effect and benefit to arterial stiffness [21]. GLP1As have been shown to slow the progression of atherosclerosis [22].

In addition to a reduction in circulating LDL cholesterol, total cholesterol, and triglycerides [23,24], GLP1As have been shown to decrease the levels of apoB48, a marker of chylomicron particles [25,26]. Recent research has indicated that GLP1As reduce intestinal chylomicron output, thus inhibiting lipid absorption [27]. GLP1As regulate intestinal lipoprotein metabolism [27,28,29].

After crossing the blood–brain barrier, GLP1As regulate lipid metabolism by activating GLP-1 receptors in the central nervous system, thus reducing chylomicron production. There may be a connection between dementia and dysregulated cholesterol metabolism, as lowering LDL cholesterol levels has been associated with potential improvements in cognitive function and dementia pathology [30,31,32].

## 4. GLP1As and Major Cardiovascular Events

Discussions and research on the cardiovascular benefits of GLP1As have been extensive and ongoing. Numerous prospective randomized controlled trials have evaluated the cardiovascular effects of GLP1As.

In a landmark trial, Marso et al. randomly assigned patients to receive semaglutide or a placebo over 104 weeks, followed by an estimation of the relative risk of major cardiovascular outcomes such as cardiovascular death, nonfatal myocardial infarction, or nonfatal stroke. The investigators found that in patients with type 2 diabetes and considered to be at high cardiovascular risk, the incidence of all the aforementioned events fell with semaglutide use [3]. Major cardiovascular outcomes were also studied in 17,604 patients 45 years or older with a BMI greater than 27, who were randomly assigned to receive 2.4 mg of semaglutide or a placebo. All these patients had cardiovascular disease but no diabetes and were placed on GLP1As. Once again, at a mean follow-up of 39.8 months, GLP1As reduced the incidence of death from cardiovascular causes, nonfatal myocardial infarction, or nonfatal stroke [33].

In another double-blind trial, Liraglutide Effect and Action in Diabetes: Evaluation of Cardiovascular Outcome Results (LEADER), a total of 9340 patients with type 2 diabetes and high cardiovascular risk were randomized to receive liraglutide or a placebo, and patients were followed for 3.8 years. The primary composite variable was the first occurrence of death from cardiovascular causes, nonfatal myocardial infarction, or nonfatal stroke. The liraglutide group had a lower rate of the primary outcome, with a hazard ratio of 0.87. A total of 278 (6.0%) patients suffered cardiovascular-related deaths in the placebo group, compared to 219 patients (4.7%) in the liraglutide group, with a hazard ratio of 0.78. In addition, the liraglutide group had a lower overall death rate (8.2%) compared to the placebo group (9.6%), with a hazard ratio of 0.85. The incidence of nonfatal myocardial infarction, nonfatal stroke, and hospitalization for heart failure was slightly lower in the liraglutide group compared to the placebo group, but the difference was not statistically significant. The primary reason for the discontinuation of liraglutide was unacceptable gastrointestinal adverse effects. However, there was no significant difference in the occurrence of pancreatitis between the liraglutide group and the placebo group [2].

In the SUSTAIN 6 (Semaglutide Unabated Sustainability) trial, 3297 patients, who were on standard diabetes treatment, were assigned to receive either once-weekly semaglutide injection or a placebo for 104 weeks. Of the 3297 patients, 2735 (83.0%) had preexisting cardiovascular and/or chronic kidney disease. The main composite outcome was the first occurrence of cardiovascular death, nonfatal myocardial infarction, or nonfatal stroke. A total of 108 out of 1648 patients (6.6%) in the semaglutide group experienced such an outcome, compared to 146 out of 1649 patients (8.9%) in the placebo group. A total of 2.9% of patients on semaglutide had nonfatal myocardial infarction compared to 3.9% on a placebo; nonfatal stroke occurred in 1.6% and 2.7%, respectively. However, the cardiovascular death rates were comparable between both groups. The semaglutide group had lower rates of new or worsening nephropathy but surprisingly experienced significantly higher rates of retinopathy complications compared to the control group. In the LEADER trial, the semaglutide group experienced a higher number of adverse events, especially gastrointestinal, that prompted stoppage [3].

In the PIONEER 6 study (Peptide Innovation for Early Diabetes Treatment 6 study), 3183 patients were randomly given either oral semaglutide or a placebo. The average age of these patients was 66 years. Of these, 2695 patients (84.7%) were aged 50 or above and had either cardiovascular or chronic kidney disease. The trial’s midpoint time was 15.9 months. In the oral semaglutide group, 61 out of 1591 patients (3.8%) experienced major adverse cardiovascular events compared to 76 out of 1592 (4.8%) in the placebo group, with a hazard ratio of 0.79. Results for the primary outcome component included 15 out of 1591 patients (0.9%) in the semaglutide group dying from cardiovascular causes, compared to 30 out of 1592 (1.9%) in the placebo group. For nonfatal myocardial infarction, the numbers were 37 out of 1591 patients (2.3%) and 31 out of 1592 (1.9%), respectively. In terms of nonfatal stroke, the figures were 12 out of 1591 patients (0.8%) and 16 out of 1592 (1.0%), respectively. Death occurred in 23 out of 1591 patients (1.4%) who took oral semaglutide and in 45 out of 1592 patients (2.8%) who took a placebo. More patients stopped taking oral semaglutide due to gastrointestinal side effects compared to those who were taking a placebo [34].

The REWIND (Researching Cardiovascular Events With a Weekly Incretin in Diabetes) double-blind, randomized placebo-controlled trial recruited 9901 participants, with an average age of 66.2 years and a median HbA1c of 7.2%. These type 2 diabetics had either a previous cardiovascular event or cardiovascular risk factors. They were randomized to receive either dulaglutide (4949) or a placebo (4952). Over a median follow-up time of 5.4 years, 594 (12.0%) participants in the dulaglutide group experienced the primary outcome (defined as the first occurrence of the composite endpoint of nonfatal myocardial infarction, nonfatal stroke, or death from cardiovascular causes) at a rate of 2.4 per 100 person-years, while that number was 663 (13.4%) (2.7 per 100 person-years) in the placebo group. No significant difference in overall mortality between the dulaglutide group (536 [10.8%]) and the placebo group (592 [12.0%]) was noted. A total of 2347 (47.4%) participants who took dulaglutide experienced a gastrointestinal adverse event during the follow-up period, while 1687 (34.1%) of those who took a placebo had the same issue [4].

Giugliano et al. conducted a meta-analysis of many large randomized controlled trials and concluded that GLP1As reduce major adverse cardiovascular event (MACE) risk in T2DM patients by 14% in comparison to a placebo [35]. The three components of MACE, namely cardiovascular mortality, nonfatal strokes, and nonfatal heart attacks, decreased, respectively, by 13%, 16%, and 9%. The decrease in risk was not influenced by the composition of the medications, regardless of whether they were exendin-4-based or human equivalents. Furthermore, Giugliano et al. found that these drugs lowered the risk of MACE more in patients with preexisting heart ailments than those without. D’Andrea et al. found a comparable result, with patients having preexisting CVD experiencing a 14% decrease in MACE on GLP1As, while those at high CVD risk but no prior events showed little to no impact [36].

Kristenson et al. performed another meta-analysis of cardiovascular outcome trials involving GLP1As. They included the following 7 trials: ELIXA (lixisenatide), LEADER (liraglutide), SUSTAIN-6 (semaglutide), EXSCEL (exenatide), Harmony Outcomes (albiglutide), REWIND (dulaglutide), and PIONEER 6 (oral semaglutide) [2,3,4,34,37,38,39]. They concluded that GLP1A treatment reduced the incidence of MACE by 12%. There was no statistically significant heterogeneity across the subgroups examined. The risks of death from cardiovascular causes, fatal or nonfatal stroke, and fatal or nonfatal myocardial infarction were all reduced. GLP-1 receptor agonist treatment reduced all-cause mortality by 12%, hospital admission for heart failure by 9%, and a broad composite kidney outcome (development of new-onset macroalbuminuria, decline in estimated glomerular filtration rate [or increase in creatinine], progression to end-stage kidney disease, or death attributable to kidney causes) by 17%, mainly due to a reduction in urinary albumin excretion. There was no increase in the risk of severe hypoglycemia, pancreatitis, or pancreatic cancer [40].

In a systematic review and meta-analysis, Alexander et al. primarily examined the lasting advantages and drawbacks of GLP1As on cardiovascular health. Their findings give confidence that the decrease in major adverse cardiovascular events was in fact attributable to GLP1As [41].

A major limitation of the above studies (and the resulting meta-analyses) is that they all received grants from the drug manufacturer. The potential bias involved in the conduct and reporting from such trials should not be ignored [42,43,44].

## 5. GLP1As and Non-Alcoholic Steatohepatitis

Currently, there is a notable rise in the number of individuals diagnosed with non-alcoholic fatty liver disease (NAFLD), with a prevalence rate as high as 38% in the USA. Among people with type 2 diabetes, the prevalence is 55–70% [45]. It has exceeded viral liver disease to become the most prevalent liver disease globally, prompting the need for additional research. Across the world, 1 out of every 25 deaths are liver related [46].

Numerous studies indicate that persistent NAFLD predisposes an individual to additional serious conditions such as liver cancer and liver failure. NAFLD is categorized into non-alcoholic fatty liver (NAFL) and non-alcoholic steatohepatitis (NASH), with a myriad of clinical symptoms and varying rates of progression [47]. At the heart of the problem is the accumulation of fat in the hepatocytes in the presence of excessive metabolic substrates, leading to the gradual production of potentially harmful lipids and a rise in de novo lipogenesis [48]. NASH is associated with hepatocellular inflammation and destruction and is considered a more severe type of NAFLD, which may lead to cirrhosis and liver cancer [49]. Currently, there are no effective treatment options.

Obesity and type 2 diabetes are two important risk factors for NASH [50]. In addition to liver damage, NASH increases the potential for cardiovascular diseases [5]. Insulin resistance in the liver and adipose tissue is seen as a key factor in the severity of NASH and its associated risks. GLP1As assist in blood sugar management and weight reduction, as well as stimulate liver enzymes in individuals with type 2 diabetes. The beneficial biochemical effects of GLP1As make them an attractive option for diabetic management in patients with NAFLD. After the administration of liraglutide for 26 weeks in patients with type 2 diabetes, Armstrong et al. demonstrated an improvement in liver enzymes in patients, probably mediated by its action on weight loss and glycemic control [6]. Of the 4442 patients they analyzed (from the ‘Liraglutide Efficacy and Action in Diabetes’ program [51,52,53,54,55,56]), 2241 (50.8%) patients had abnormal alanine transferase at baseline. The administration of 1.8 mg of liraglutide was associated with a reduction in alanine aminotransferase (in comparison to a placebo). Newsome et al. in a 72-week, double blind, phase 2 trial involving patients with biopsy-confirmed NASH and liver fibrosis found that semaglutide resulted in a significantly higher percentage of patients with NASH resolution than the placebo [57]. As a result, GLP1As could be an effective tool to manage both diabetes mellitus and NAFLD [58].

## 6. GLP1As and Associative Learning

One of the ways we learn is by observing connections or associations. In this process, we can glean new information using the old information already etched in our memory. It is said that associative learning refers to the processing of sensory information to best suit our physiological needs. Zhang demonstrated impairment in reward-based associative learning specific to food in obese women. A robust negative association between body mass index and learning performance in the food domain is also seen in women [9]. Associative learning is shown to improve with liraglutide in patients with obesity [59].

The midbrain region that releases dopamine is important for learning appropriate behaviors [60] and is especially affected by signals from the body’s metabolism and gut hormones such as GLP-1. In a single-blinded, randomized, controlled, crossover study using human functional magnetic resonance imaging, Hanssen et al. classified study participants based on insulin sensitivity levels. In this computational model of behavioral response learning, the investigators demonstrated that impaired metabolic sensing in obesity reduces adaptive learning. They also showed that liraglutide restores dysfunctional sensory association learning in the obese group. The activation of GLP-1 receptors influences associative learning in individuals with obesity by modulating the mesoaccumbens pathway in the brain [59].

## 7. GLP1As and Alzheimer’s Dementia

While medical advances have prolonged the human lifespan, the resulting burden of dementia is affecting every part of society and is being addressed on a war footing. Among Americans aged 65 and over, nearly 6.5 million have Alzheimer’s dementia (AD), and unless the disease can be effectively treated or prevented, this number is likely to reach 13.8 million by 2060 [61]. Although the affected individual is, in some ways, oblivious to the worsening memory impairment, the burden it places on family, friends, caregivers, and voluntary and government organizations is staggering. Yearly spending by the federal government on AD and all other dementia research has reached 3.8 billion US dollars [62]. In terms of medications, efforts are directed towards both creating new molecules and exploring the benefits of existing drugs used for other indications. GLP1As belong to the second group.

Recent studies, both experimental and clinical, have indicated that AD could be classified as a metabolic disorder related to T2DM, also known as type 3 diabetes in certain cases [63]. A post-mortem investigation has shown that insulin resistance is present in the brains of patients with Alzheimer’s disease, along with, as the disease advances, a notable reduction in insulin receptor expression. There are indications of defects in insulin signaling associated with AD [64,65,66]. Epidemiological research has discovered links between T2DM and AD [67].

At the 2024 Alzheimer’s Association International Conference, researchers from Imperial College London, United Kingdom, stated that “liraglutide may protect the brains of people with mild Alzheimer’s disease and reduce cognitive decline by as much as 18% after one year of treatment compared to placebo, by slowing the shrinking of the parts of the brain that are vital for memory, learning, language and decision-making” [68].

Baker et al., from Veterans Affairs Puget Sound Health Care System, Seattle, WA, USA, demonstrated that increased insulin resistance (responsible for prediabetes and T2DM) is associated with reductions in the cerebral glucose metabolic rate (CMRglu). Such reductions in the parietotemporal, frontal, and cingulate cortices of the brain is associated with increased AD risk, and these changes are observed many years before the onset of dementia. They concluded that insulin resistance may be a marker of AD risk, which in turn is associated with reduced CMRglu and subtle cognitive impairments at the earliest stage of the disease, even before the onset of true cognitive impairment [69].

Animal models have suggested that once-daily subcutaneously administered liraglutide may alleviate cognitive impairment in AD by decreasing the phosphorylation of tau [70]. After 8 weeks of treatment, liraglutide prevented memory impairments in the Y Maze and Morris Water Maze following experimentally induced AD in mice. Recent experimental studies also suggest that the majority of injected GLP1 analogs are present in the brain, suggesting that they penetrate the blood–brain barrier and can have direct physiological effects on the brain [71].

Based on the aforementioned experimental findings, it can be deduced that incretin-related drugs used for diabetes treatment could have an indirect impact on Alzheimer’s disease. It has been shown that liraglutide can decrease AD pathologic markers, such as oligomeric Aβ and Aβ plaque load, in the APP/PS1 mouse model, leading to reduced microglial activation and improved memory behaviors [72]. A meta-analysis of 26 studies evaluated the effects and potential mechanisms of GLP1As in AD animal models [73]. The results showed that GLP1As could improve the learning and memory in rodents with induced AD.

Furthermore, mice with increased levels of GLP1A in the hippocampus displayed enhanced neurite growth and better spatial learning skills. A randomized, placebo-controlled, double-blind study has shown that liraglutide can enhance glucose metabolism and cognition in patients with Alzheimer’s disease [74,75].

## 8. GLP1As and Parkinsonism

Similar to Alzheimer’s dementia, Parkinson’s disease is a common neurodegenerative illness typically affecting the elderly. Together, they affect nearly 8 million people in the US alone [76]. The loss of dopaminergic neurons in the substantia nigra pars compacta region of the midbrain is the chief histologic finding in patients with Parkinson’s disease. This results in a decrease in dopamine levels in the striatum. Recently it is understood that insulin resistance could be a central factor in the development of Parkinson’s disease. A link between inflammation and certain Parkinson’s disease symptoms is demonstrated, and such markers of inflammation are seen in their blood and cerebrospinal fluid samples [77].

Protein kinase B (referred to as Akt) is a serine/threonine kinase and one of the three protein kinases that are important in many cellular processes [78]. Insulin encourages glucose transport activity through two pathways: a contraction-stimulated pathway that depend on Ca2+/5′-monophosphate-activated protein kinase (MAPK) and an insulin-dependent pathway that is activated via the upregulation of Akt [79]. The second pathway is compromised in patients with type 2 diabetes. Exenatide, a GLP1A, is found to restore insulin signaling in neurons by restoring Akt signaling and suppressing MAPK pathways. GLP-1R agonism can reverse neuronal toxicity linked to synucleinopathy by decreasing oxidative stress, enhancing mitochondrial and lysosomal function, reducing α-synuclein aggregation, and improving neuronal survival. The synucleinopathies consist of heterogenous neurodegenerative disorders, and all of them exhibit aggregates of the insoluble α-synuclein protein in selectively vulnerable populations of neurons and glia [10]. The administration of GLP1A is shown to reduce inflammatory reactions caused by synuclein in glial cells, resulting in neuroprotection via indirect effects on other cells.

By using human-induced pluripotent stem cell (iPSC) models of synucleinopathy, Athauda et al. demonstrated that those with higher baseline MAPK expression (and therefore insulin resistance) showed an improvement with exenatide treatment. Exenatide therapy resulted in decreased α-synuclein clumps and decreased levels of the inflammatory cytokine IL-6. Their study demonstrated the basis of the potential benefits of GLP-1R agonism in neurons and astrocytes, and it pinpoints who is most likely to gain from GLP-1R agonism and underscores the effectiveness of GLP-1R agonism as a strategy to modify diseases in synucleinopathies.

In a single-center, placebo RCT, 63 participants were either assigned to receive liraglutide or a placebo. At 54 weeks, there was a 6.6-point improvement in non-motor symptoms scale scores for the liraglutide group and a 6.5-point deterioration in the placebo group, resulting in a 13.1-point adjusted mean difference. Adverse events were frequent with injection site reactions and gastrointestinal symptoms. There were eleven serious adverse events documented, all of which were unrelated to the intervention being studied [80].

New research from Zhang et al. has explored how the GLP-1 analogs semaglutide and liraglutide provide protection for the brain in the chronic 1-methyl-4-phenyl-1,2,3,6-tetrahydropyridine (MPTP) mouse model of Parkinson’s disease. MPTP is a neurotoxin that can induce Parkinson’s disease-like symptoms in humans and animal models. Both semaglutide and liraglutide improved motor impairments induced by MPTP. Furthermore, both medications also increased the levels of tyrosine hydroxylase, decreased α-syn accumulation, relieved brain inflammation, lowered lipid peroxidation, blocked the mitophagy pathway in the mitochondria, and raised glial cell line-derived neurotrophic factor expression, which safeguards dopaminergic neurons in the substantia nigra and striatum. Zhang et al. found that the long-acting GLP-1 analog semaglutide showed greater potency in comparison to once-daily liraglutide across most parameters analyzed. The authors concluded that semaglutide could be a potentially effective treatment in patients with Parkinson’s disease [81].

## 9. GLP1As and Stroke

The data regarding the effectiveness of GLP1As in this area are limited. A recent study reported that GLP1A treatment attenuated the onset of stroke. Another study demonstrated that GLP1A administration reduced stroke volume, improved neuronal survival, and suppressed neurologic deficit [82,83]. Liraglutide reduced the levels of vascular cell adhesion molecule 1 and E-cadherin in endothelial cells, leading to a decrease in hypertension and stroke. The GLP-1 agonist exendin-4 also decreased blood cell infiltration and adhesion in the atherosclerosis model [84,85,86].

New studies indicate that both GLP1As and DPP-4 inhibitors may serve as regulators against strokes and lower the frequency of strokes in clinical settings. DPP-4 inhibitors and GLP-1R agonists may prevent strong inflammatory reactions in monocytes and macrophages, leading to a reduction in atherosclerotic lesion advancement in apoE-knockout mice [87,88,89]. GLP1As improved the function of endothelial cells and reduced the expression of cell adhesion markers in blood vessels in a knockout animal model. GLP1As shielded the BBB by reducing the expression of matrix metallopeptidase 9 and decreased vascular permeability by blocking intercellular adhesion molecule 1 expression [90,91,92].

## 10. GLP1As and Cancer

Although studies have documented the benefits of GLP1As in cancer, the data are mixed. In a small recent study of 30 patients with cholangiocarcinoma (CCA), it was detected that GLP-1 receptor expression in CCA tissues was associated with poor histological grading. The administration of liraglutide appeared to downregulate these receptors and decrease the migration of CCA cells [93].

Over a 15-year period, in a study of 1,221,218 drug-naive patients with T2D, it was found that GLP1As reduced the risk of colorectal cancer compared to insulin, metformin, SGLT2 inhibitors, sulfonylureas, and thiazolidinediones. The risk was also lower when compared to alpha-glucosidase or DPP-4 inhibitors; however, the difference was not statistically significant. Similar results were seen in both women and men. GLP1As were linked to a reduced chance of colorectal cancer in obese/overweight patients versus insulin, metformin, and/or other diabetes medications [8].

Patients receiving insulin compared to those on GLP1A treatment had significantly reduced risks of many other cancers including meningioma, pancreatic, ovarian, multiple myeloma, esophageal, endometrial, renal, and hepatocellular [94].

Alternatively, in a database study of 2562 case subjects, patients exposed to a GLP1A for 1 to 3 years displayed a 58% higher risk of all thyroid carcinomas and a 78% higher risk of medullary thyroid cancer. Patients in the remaining two exposure groups (≤1 year and over 3 years) also exhibited a higher risk of developing thyroid cancer, but the difference between the groups was not statistically significant due to a small number of cases [95].

Liraglutide has been shown to increase the risk of thyroid, pancreatic, and early breast cancer in animals. It is unclear if this applies to humans. The potential increased risk of cancer was investigated by Jujic et al. while investigating the relationship between endogenous incretin levels and incident cancer [96]. In addition to finding that incretins stimulate cellular proliferation, they noted an increased risk of thyroid and pancreatic cancer with GLP1A usage. Other studies have reported an increased risk of pancreatic cancer associated with GLP1A usage [50,97], while some did not find such an association [98,99]. The observed associations are often attributed to an occult pancreatic cancer that provokes or aggravates diabetes [100].

## 11. GLP1As and PCOS and Fertility

Polycystic ovary syndrome is associated with many metabolic implications such as obesity, insulin resistance, impaired glucose metabolism, dyslipidemia, hypertension, metabolic syndrome, and an increased risk of diabetes. Szczesnowicz et al. state that GLP1As can regulate insulin release, decrease hyperglycemia, and improve insulin resistance, as well as produce weight loss. As a result, GLP1As can have profound effects on weight loss and fertility in patients with PCOS [101]. It has also been well established that controlled weight loss can improve fertility in patients. Clark et al., in a study of 67 infertile patients, noted that over a 6-month period, participants lost an average of 10 kg. They found that ovulatory function was restored in 60 women (90%), of whom 52 (78%) conceived with a miscarriage rate of 18% [102].

Short-term weight loss is also beneficial in assisted reproduction. Chavarro et al. found that short-term weight loss is associated with increased metaphase 2 oocyte retrieval [103]. However, they did not find a positive association with pregnancy rates or live birth rates. Kort et al. found that conception rates and live birth rates were increased in patients who underwent 10% or more weight loss [104].

In women with PCOS, GLP1As can cause significant weight reduction, which in turn can also decrease testosterone levels, improve reproductive function, decrease hyperlipidemia, and improve glycemic control, as well as hypertension [105].

In a meta-analysis and systematic review involving 11 RCTs with 840 patients, Zhou et al. found that GLP1As enhanced the rates of natural pregnancy and improved menstrual regularity. There were no significant statistical variances in the overall pregnancy rate and the IVF pregnancy rate between the two groups; however, the total pregnancy rate increased shortly after GLP1Ras, based on subgroup analysis. Randomization to treatment with GLP1RAs led to a significant improvement in homeostasis model assessments for insulin resistance, body mass index, waist circumference, and sex hormone binding globulin, and a small decrease in total testosterone was observed when compared to the control group [11].

In men, those with T2DM and obesity noticed improved morphology of sperms after taking semaglutide. In an open-label trial involving 25 men with T2DM, who were randomized to semaglutide 1 mg/week or intramuscular testosterone undecanoate, the former group had a significantly higher number of morphologically normal sperm, sperm concentration, and total number. They also noticed marked improvements in total testosterone levels and symptoms of hypogonadism [106].

## 12. GLP1As and Mental Health

The activation of GLP1 receptors in the mesolimbic pathways in the brain is responsible for the inhibition of the hedonic value of many addictive triggers. Arillota et al. studied the effects of GLP1As on substance and behavioral addictions, including alcohol, caffeine, nicotine, cannabis, psychostimulants, compulsive shopping, and sex drive/libido [107]. Using a web application, they analyzed online discussions from multiple social media platforms such as YouTube, Reddit, and TikTok. They found that 29.75% of alcohol-related, 22.22% of caffeine-related, and 23.08% of nicotine-related comments clearly stated a cessation of the intake of these substances following the start of GLP1As. A total of 21.35% of comments reported a compulsive shopping interruption, whilst sexual drive/libido increased in several users. In a landmark study in *Nature*, of the 83,825 patients with obesity, who had no prior diagnosis of alcohol use disorder (AUD) and were for the first time prescribed semaglutide or non-GLP1A anti-obesity medications including naltrexone or topiramate, it was found that compared to non-GLP1RA anti-obesity medications, semaglutide was associated with a significantly lower risk of recurrent AUD diagnosis, a result consistent across gender, age group, and race [108]. Significant lower risks were observed in patients with and without T2DM. As there are reports of positive appetitive behavior changes with GLP1As, many have suggested caution when considering the drug in patients with increased risk of suicidality [109]. McIntyre et al. compared suicidality associated with all GLP1As relative to other glucose-lowering agents currently approved by the United States Food and Drug Administration [110]. They studied reports of suicidal ideation, suicidal behavior, suicidal attempts, and completed suicide associated with GLP1A exposure reported to the FDA between 2005 and October 2023; data were obtained from the FDA Adverse Event Reporting System (FAERS). They concluded that although there was a disproportionate reporting of suicidal ideation and the “depression/suicidal” category with semaglutide and liraglutide, there was no increased reporting of suicidal behavior, suicide attempts, and completed suicide of any of the FDA-approved GLP1As. Moreover, patients with schizophrenia have five times the prevalence of diabetes and obesity, contributing to a 20-year reduced lifespan, and a recent study has shown that there may be benefits to reducing the weight gain induced by antipsychotics via GLP1As [111]. Trott et al., in their review, have suggested that the initiation of GLP1As at the time of antipsychotic treatment commencement (particularly in first-episode psychosis) could minimize, if not prevent, antipsychotic weight gain entirely, and thus prevent decreases in quality of life while supporting medication adherence. However, more double or triple agnostic studies are needed to confirm the results [112].

## 13. GLP1As and Chronic Pain

Nozu et al., in a recent study in rats, found that liraglutide blocked lipopolysaccharide-induced visceral allodynia. A possible mechanism could be the inhibition of the production of pro-inflammatory cytokines. Moreover, GLP1R activation on microglia results in the activation of the cAMP/PKA/p38β/CREB signal transduction pathway, promoting the expression of IL-10. On subsequent autocrine activation of the IL-10 receptor/STAT3 signaling pathway in microglia, there is enhanced expression and release of β-endorphin. This acts on μ-opioid receptors on neurons to produce analgesic and neuroprotective effects [113,114]. In another study in rats, published in *Nature*, Go et al. found that GLP-1-derived peptides (liraglutide, exendin-4, and exendin 9–39) inhibited capsaicin (CAP)-induced currents and calcium responses in cultured sensory neurons and TRPV1-expressing cell lines. They found that exendin decreased CAP-induced acute pain, as well as chronic pain induced by complete Freund’s adjuvant (CFA) and spared nerve injury (SNI) in mice without causing hyperthermia associated with other TRPV1 inhibitors [115]. However, more studies are needed to confirm similar effects in humans. In a study published in the *Journal of Orthopaedic Translation*, Meurot et al. postulated that incretinomimetics have beneficial pleiotropic effects such as immunomodulation, anti-inflammation, and neuronal protection and may slow joint structural changes [116]. They stated that patients receiving a GLP1A had greater improvements in pain and function than patients treated with weight loss alone. However, many of these studies are industry sponsored and more unbiased studies with randomization and a larger sample size are needed to prove the same results.

## 14. GLP1As and COVID-19

GLP1As have pleiotropic effects that blunt the systemic inflammatory response. They inhibit cytokine release due to interferences with Nf-kB signaling pathways [117]. Steven et al., in their study in animal models, found that the administration of liraglutide in lipopolysaccharide-induced endotoxemia in animal models has been shown to improve survival and vascular dysfunction, along with salubrious actions on inflammatory and hemostatic parameters [118]. Thrombotic events are increased in COVID-19 patients and form one of the major causes of mortality [119]. Sternkopf, in their study of native GLP1, proved that venous and arterial clots may be decreased with GLP1As [120]. Scirica et al., in their landmark SELECT (Semaglutide Effects on Cardiovascular Outcomes in Patients With Overweight or Obesity) study, found that semaglutide did not reduce incident COVID-19. However, among participants who developed COVID-19, fewer participants treated with semaglutide had COVID-19–related serious adverse events or died of COVID-19. GLP1As can cause a decrease in obesity, which is an independent risk factor for COVID-19 mortality. Semaglutide is seen to decrease COVID-19-related cardiovascular deaths as well [121].

## 15. Conclusions

The information presented in this review discusses the wide-ranging benefits of GLP1 agonists well beyond their indicated uses. Although some benefits appear to be less convincing, evidence of others such as cardiovascular benefits are more definitive. Connecting the benefits to the cellular and biochemical changes is more likely to persuade skeptical readers. Although there could be hesitation in prescribing these drugs for non-diabetic benefits presently, a case can be made for a preference to GLP1As in diabetics with a coexisting predisposition to other diseases. GLP1As could become first line or part of an antidiabetic drug combination if patients have a strong family history of cardiovascular disease or neurodegenerative condition predisposition. Further research would allow clinicians to make an informed decision in presenting options to their patients with regard to GLP1 agonists, in both diabetics and non-diabetics.

## Figures and Tables

**Table 1:** Conditions strongly associated with diabetes and obesity.

Condition	Study Type	Number of Participants	Agent	Outcomes	Adverse Effects	Reference
Hypertension	SURMOUNT-1: Randomized, placebo-controlled trial	600 participants	Tirzepatide	All doses reduced 24 h systolic BP at 36 weeks compared to placebo	Nausea, vomiting	[1]
Major adverse cardiovascular events	LEADER Study: Double-blind trial	9340 patients with T2DM and high CV risk	Liraglutide	Liraglutide group had a lower primary outcome rate (13.0% vs. 14.9% in placebo), HR = 0.87	Nausea, vomiting	[2]
	SUSTAIN-6 Trial	3297 patients on standard diabetes treatment	Semaglutide	Major CV events in 6.6% of semaglutide group vs. 8.9% of placebo	Nausea, vomiting	[3]
	REWIND Trial: Double-blind, randomized, placebo-controlled	9901 participants	Dulaglutide	MACE events: 12% (dulaglutide) vs. 13.4% (placebo)	GI intolerance	[4]
Non-alcoholic steatohepatitis (NASH)	Meta-analysis of 6 RCTs	4442 patients (2241 with abnormal ALT)	Liraglutide	Reduced aminotransferase levels vs. placebo	Nausea, vomiting	[5,6]
Stroke	Meta-analysis of 8 RCTs	56,251 patients	GLP1As	Reduced non-fatal stroke and total strokes by 16%	Nausea, vomiting	[7]
Cancer	15-year cohort study	1,221,218 patients with T2DM	GLP1As	GLP1As reduced colorectal cancer risk vs. insulin, metformin, and other diabetes drugs	—	[8]

**Table 2:** Conditions not commonly associated with diabetes and obesity.

Condition	Study Type	Number of Participants	Agent	Outcomes	Adverse Effects	Reference
Associative learning	Single-blind RCT	30 participants with normal; 24 participants with impaired insulin sensitivity	Liraglutide	Restored sensory association learning in obese individuals	Nausea, vomiting	[9]
Parkinsonism	Single-center, placebo-controlled RCT	63 patients	Liraglutide	6.6 point improvement in liraglutide group and 6.5 point deterioration in placebo group	Nausea, vomiting	[10]
PCOS andFertility	Meta-analysis of 11 RCTs	840 patients	GLP1As	Increased natural pregnancy rates and improved menstrual regularity	Nausea, vomiting	[11]
